# A high sensitivity, low cost and fully decoupled multi-axis capacitive tactile force sensor for robotic surgical systems

**DOI:** 10.1371/journal.pone.0313737

**Published:** 2024-11-19

**Authors:** Sajid Hussain, Muhammad Mubasher Saleem, Muhammad Rehan, Hassan Elahi, Mohsin Islam Tiwana

**Affiliations:** 1 Micro/Nano Robotic Technologies Lab, Department of Mechatronics Engineering, National University of Sciences and Technology, Islamabad, Pakistan; 2 National Centre of Robotics and Automation, Islamabad, Pakistan; National Research Centre, EGYPT

## Abstract

This paper presents the design of a multi-axis capacitive tactile force sensor with a fully decoupled output response for input normal and shear forces. A patterned elastomer is used as a dielectric layer between capacitive electrodes of the sensor that allows to achieve relatively higher sensitivity. The sensor is fabricated utilizing a low-cost rapid prototyping technique and is characterized for normal and shear forces in the range of 0 ~ 10 N and 0 ~ 3.1 N respectively. The achieved force sensitivity for the normal axis is 2.03%/N and for shear axes is 1.67%/N. The difference between the estimated force from the sensor and actual force applied is negligible, which demonstrates the accuracy of the sensor. The reliability of the sensor is analysed by performing hysteresis and repeatability tests. The hysteresis error is found to be 4.94% and 4.69% for normal and shear forces respectively. The repeatability error of the sensor is less than 5%, which shows the stability of the sensor. The high sensitivity, linear output response, high force measurement range, reliability and low cost make the proposed tactile sensor suitable for the force feedback in the robotic surgical systems.

## Introduction

In minimally invasive robotic surgery (MIRS) utilizing telemanipulation, a surgeon’s hand movements are translated into corresponding manipulation of the surgical instruments inside the human body. Hosseinabadi and Salcudean [1] identified that a key requirement of MIRS is enabling the surgeon to perform tissue palpation and texture assessment through haptic and tactile force feedback. This is typically accomplished using tactile sensors integrated into the surgical tools, which measure various properties such as force, pressure, vibration, temperature, softness, shape, and texture, providing the surgeon with a sense of palpation and touch. Stassi et al. [2] outlined the challenges in developing tactile force sensors for robotic surgical systems, which include achieving high sensitivity, high resolution, wide force range, low hysteresis, repeatability, and low power consumption. Recent research has focused on designing tactile sensors for a wide range of applications including human-robot interaction (Cirillo et al. [3], Pang et al. [4] and Zhao et al. [5]), wearable and artificial skins (Liu et al. [6] and Rado et al. [7]), healthcare and biomedical engineering (Nag et al. [8], Roy et al. [9] and Rehan et al. [10]). Different transduction mechanisms—such as piezoelectric, piezoresistive, magnetic, capacitive, and optical—are commonly employed in tactile force sensor design. Zhu et al. [11] extensively discussed the advantages and limitations of each.

The capacitive transduction mechanism is most widely used for the design of tactile sensor due to its relatively simple construction and working principle, temperature independent response, high sensitivity and resolution, high sensor to noise ratio (SNR) and low hysteresis errors. Lee et al. [12] reported a three-axis capacitive tactile force sensor which can decouple the applied force into normal and shear direction with a sensitivity of 1.3%/mN in normal and 1.2% /mN in shear direction. The sensor is fabricated using MEMS technology with a size of 2mm × 2mm and can measure input forces in the range of 0 ~ 20 mN in both normal and shear axis. Dobrzynska and Gijs [13] proposed a three-axis force sensor consisting of finger shaped electrode capacitors at microlevel. The sensitivity of the sensor in normal direction is 0.024 /kPa for input pressure less than 10 which decreases to 0.00066 /kPa for higher input pressure values. The shear axis sensitivity is reported to be 0.00028 /kPa for input pressure up to 220 kPa. A capacitive tactile force sensor design of 4mm × 4mm size has been presented by Liang et al. [14], that measures normal and shear forces. The input force measurement range for both normal and shear axis is 0 to 0.5 N with a normalized force sensitivity of 0.583%/N. The sensor is realized using customized microfabrication process techniques. The microscale capacitive force sensors generally have low input force measurement ranges and are realized through MEMS technology based customized relatively complex and high-cost microfabrication process. Yao et al. [15] reported a three-axis tactile force sensor which is fabricated using low cost three-dimensional (3D) printing technology and can measure input forces up to 0.7 N in normal and 0.6 N in shear axis. The reported force sensitivity values for the normal and shear axis are 1.08%/N and 1.2%/N respectively and the sensor diameter is 19.95 mm. A tri-axis capacitive force sensing array is presented by Fernandes et al. [16] with a unit cell having two parallel plates fabricated using a low-cost rapid prototyping technique. A patterned elastomer layer is used between two parallel electrodes for translational displacement of the top electrode. The sensor showed good sensitivities of 0.04%/N and 0.036%/N for normal and shear force respectively for an input force up to 15 N. Zhu et al. [17] presented a multi-axis capacitive tactile force sensor with a size of 22 mm × 22mm and input force measurement up to 50 N in normal and 10 N in shear axis. The sensor has high resolution and large measurement range with average sensitivities of 0.0028%/N and 0.0061%/N in normal and shear axis respectively. Arshad et al [18] used fringe effect of electric field for multi-axis tactile force sensing. A sensitivity of 0.0378/N for normal and 0.018/N for shear force is achieved and sensor can operate for a dynamic input of up to 3 Hz. Morton et al. [19] utilized flexible printed circuit board to make a capacitive sensor. They investigated the use of the sensor in curved geometry. The sensor exhibited output linearity with average sensitivity of 0.47%/N in normal and 1.8%/N in shear axis. Sarwar et al [20] proposed a capacitive sensor that can measure shear and normal force. They achieved a crosstalk of 2.5% between normal and shear and 10% among shear axis with normal and shear sensitivity of 0.49 kPa and 0.31 kPa. Triboelectric nanogenerators (TENGs) have also been employed in sensing applications. Hajra et al. [21] investigated the use of TENGs for various robotic applications, demonstrating a sensitivity of 1.52 mV/Pa in pressure sensor arrays. Panda et al. [22] explored the use of TENGs to measure the gripping force in robotic grippers. In another study, Hajra et al. [23] developed a TENG based on ZIF-67 for energy harvesting, showcasing the device’s ability to utilize the generated energy for both sensing and actuation in robotic grippers.

The structural designs of both MEMS and rapid prototyping-based tactile force sensors, as discussed earlier, typically utilize a square-shaped configuration with four electrodes. This configuration is intended to effectively decouple the applied normal and shear forces. However, a significant limitation of this design is that the calculation of force for a specific axis relies on the outputs of all four electrodes. This interdependence can introduce errors in measurements, as fluctuations in one electrode’s output may impact the calculated forces for the other axes. Consequently, this complexity necessitates the implementation of relatively intricate analytical sensing models to accurately decouple the input forces. In this paper, we propose a novel design for a tactile force sensor that addresses these challenges by employing a unique configuration of electrodes. Unlike traditional designs, our approach utilizes separate force-sensing electrodes for each axis, allowing for direct measurement of normal and shear forces independently. This innovative design not only simplifies the sensing process but also enhances measurement accuracy and sensitivity. By isolating the sensing functions for each axis, our sensor minimizes the potential for cross-axis interference, thereby reducing errors and improving the reliability of the force measurements. This advancement represents a significant step forward in tactile sensing technology, paving the way for more precise applications in robotic systems and other fields that require accurate force sensing.

## Sensor design

### Working principle

**Fig 1:**
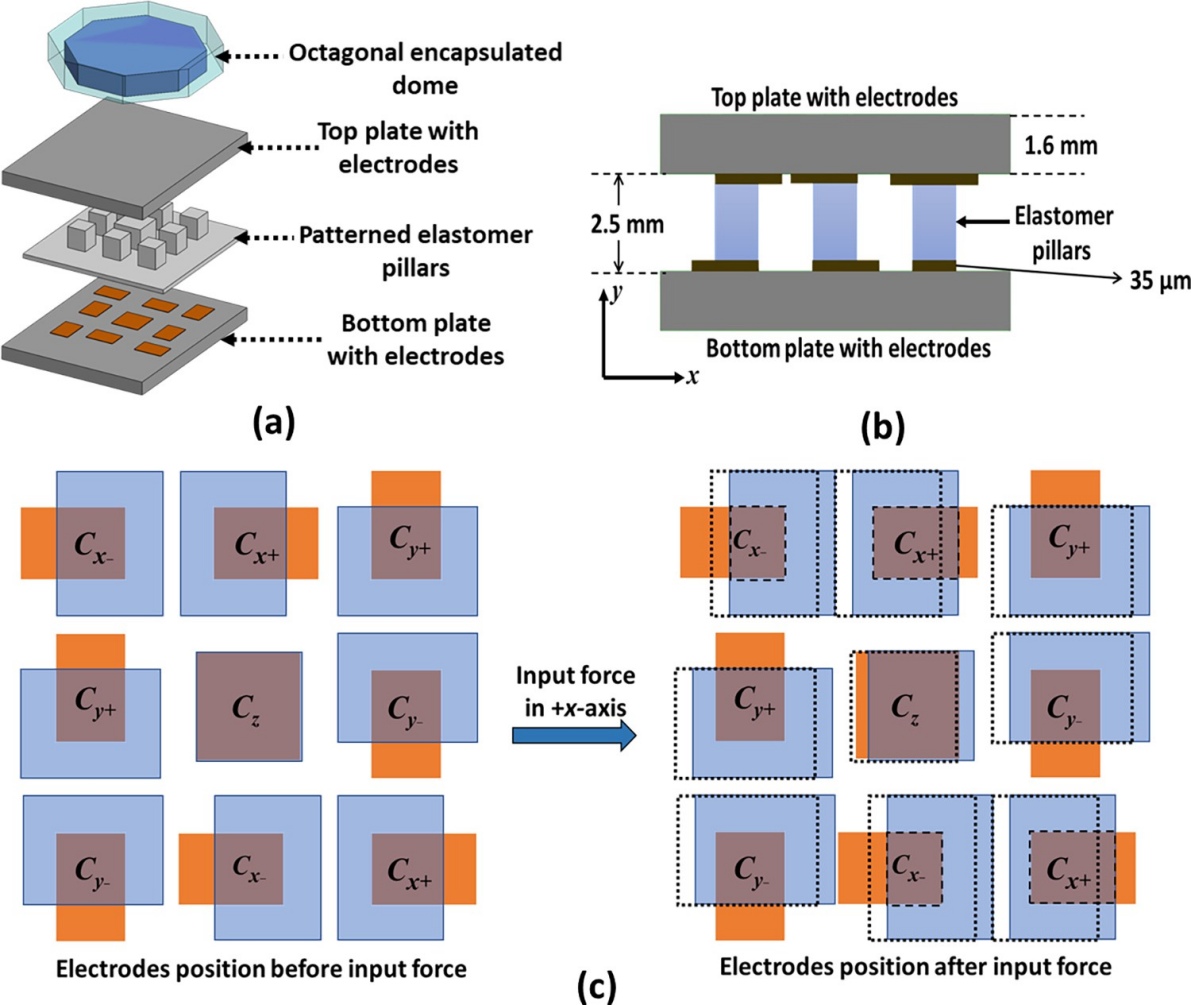
(a) Exploded view of the sensor showing different parts (b) cross-section view of the sensor (c) position of the top plate electrodes before and after application of shear force in the +x-axis.

Fig 1(A) and 1(B) show the exploded and cross-section view of the proposed tactile force sensor respectively with different parts. The sensor comprises of two parallel plates with a specific arrangement of different electrodes. The top and bottom plates are aligned such that they allow to measure both the normal and ± xy-axis shear forces, applied to the sensor. The spacing between the two parallel plates is achieved using a patterned elastomer layer consisting of pillar shaped structures with a height of 2.5 mm. The elastomer pillars are positioned such that the overlap area between the capacitive plates on the top and bottom electrodes is filled with the elastomer. The presence of elastomer between the electrodes results in an increase in the nominal capacitance of the sensor due to higher dielectric constant of the elastomer and offer low mechanical stiffness. This also makes the sensor less susceptible to humidity changes since the composition of elastomer is rarely affected by humidity. The hard material based octagonal shaped dome, encapsulated in an elastomer, at the top of the sensor is used to apply the force in both normal and shear axis during characterization.

Fig 1(C) shows the arrangement of the electrodes in both the top and bottom plate of the sensor which act as a capacitor under a bias voltage. The top and bottom plate electrodes are shown in blue and orange color respectively. The top electrodes have side dimensions of 4 mm × 3 mm while the bottom electrodes have side dimensions of 3 mm × 2 mm, The top and bottom electrodes are aligned such that the initial overlap area is 2 mm × 2 mm between all the electrode pairs excluding the center one which has an overlap area of 3 mm × 3 mm. The electrode arrangement in all shear directions is symmetric. All capacitive electrodes for capacitors associated to shear force measurement are arranged in such a way that the top electrodes are placed at an offset of 1 mm from bottom electrode. When the shear force is applied the top electrode can travel 1 mm and sensor response reaches its maximum value as the top and bottom electrodes are fully overlapped. The overall size of the sensor is 13 × 13 mm. The capacitive electrode pairs used for the measurement of an applied force in the +x-axis, −x-axis, +y-axis, −y-axis, and z-axis are labelled as *C*_*x+*_, *C*_*x−*_, *C*_*y+*_, *C*_*y−*_ and *C*_*z*_ respectively.

The sensing scheme for the proposed tactile force sensor for input force applied in different axes is shown in Table 1. The upward arrow (↑) represents increase, downward arrow (↓) represents decrease and horizontal bar (―) represents no change. For an applied force in the *z-*axis, the top plate moves downwards which results in increases in the capacitance for all the capacitive electrode pairs. The change in the capacitance value for the electrode pair *C*_*z*_ is used to measure the applied force in the normal direction. For the measurement of the shear forces in the ± *x-*axis and ± *y*-axis, the electrode pairs are arranged such that for an applied force in a specific axis, the capacitance of the electrode pair of that specific axis only increases due to change in the overlap area and remains constant or decreases for other axes, thus fully decoupling the output response. For example, for an input force in the +*x*-axis, the capacitance increases in the electrode pair *C*_*x+*_ and decreases in *C*_*x−*_ and *C*_*z*_ while remains constant for the *C*_*y+*_ and *C*_*y−*_. The capacitance of the *C*_*y+*_ and *C*_*y−*_ being unchanged for an input force in ± *x-*axis allows a fully decoupled output response of the sensor for an applied shear force.

**Table 1:** Look-up table for capacitance change corresponding to applied force in normal and shear axis.

Applied Force	Force Axis	Capacitance Change in the Electrode Pairs				
		Δ*C*_*x+*_	Δ*C*_*x−*_	Δ*C*_*y+*_	Δ*C*_*y−*_	Δ*C*_*z*_
Normal	−*z*-axis	↑	↑	↑	↑	↑
Shear	+*x*-axis	↑	↓	―	―	↓
	−*x*-axis	↓	↑	―	―	↓
	+*y*-axis	―	―	↑	↓	↓
	−*y*-axis	―	―	↓	↑	↓

The sensor’s response is completely decoupled, as shown in Fig 1(C). When a force is applied along the +x-axis, the top plate electrodes move in the +x-axis direction. Observing the electrode positions after applying the force reveals that only the overlap area of the x-axis capacitors, labeled *C*_*x+*_ and *C*_*x =*_, changes. Meanwhile, the overlap area of the y-axis capacitors, labeled *C*_*y+*_ and *C*_*y-*_, remains unaffected. This indicates that for a force applied along the x-axis, there is no change in the y-axis capacitances, confirming a fully decoupled response.

In the proposed sensor design, two electrode pairs are placed at the opposite sides of the plates for measuring the applied force in shear axis. The initial capacitance for a shear axis electrode pair, with an overlap area of *A* and elastomer height of *d*, can be written as,

$$C=C_{1}+C_{2}=\frac{2\varepsilon A}{d}$$ (1)

**Fig 2:**
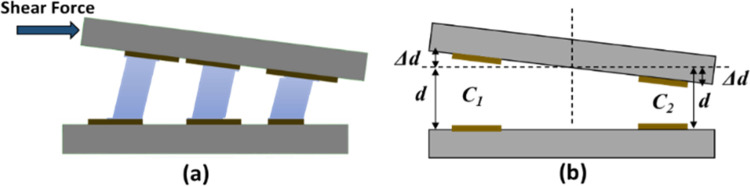
(a) the displacement in the top electrode plate for an input shear force (b) the symmetric change in gap between the top and bottom electrode plate with respect to center.

For an applied force in the shear axis, an undesired deflection in the top plate occurs due to a tilt about its own axis as shown in Fig 2A & 2B. The resultant change in the capacitance for a shear axis electrode pair can thus be written as.

$$\Delta C=\varepsilon A\left\lfloor \frac{1}{d+\Delta d}+\frac{1}{d-\Delta d}\right\rfloor =\frac{2\varepsilon A}{d}\left\lfloor \frac{1}{1+{\left(\frac{\Delta d}{d}\right)}^{2}}\right\rfloor$$ (2)

Assuming that the deflection in the top plate Δ*d* is very small as compared to the initial gap *d*, the ${\left[\frac{\Delta d}{d}\right]}^{2}$term can be ignored. Thus, the symmetric placement of two electrode pairs, about tilt axis of the top plate, nulls the change in capacitance, due to undesired deflection, for a ± *x-*axis and ± *y*-axis shear forces.

### Sensor fabrication

**Fig 3:**
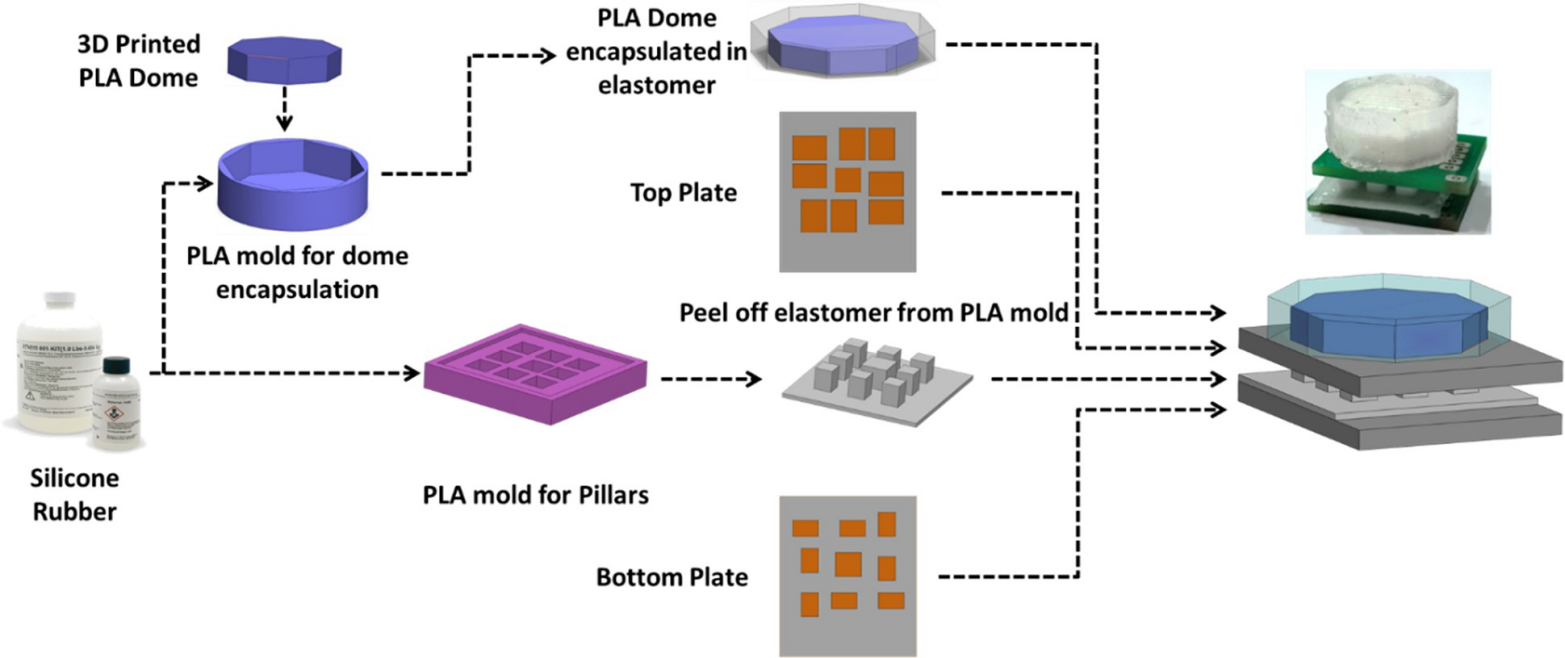
Different fabrication process steps for the proposed tactile force sensor.

Fig 3 shows the fabrication process flow for the tactile force sensor. The elastomer used for the sensor is RTV-528 silicone rubber. A 1% volume ratio of the curing agent is added to the RTV-528 silicone rubber and stirred until a fiber like threads start to form. It is then degassed and poured into 3D printed mold with patterned holes. The mold is then cured at room temperature for 24 hours to get silicone rubber pillars. The top octagonal shaped sensor dome, for the force application, is manufactured using 3D printing with polylactic acid (PLA) material. The dome is then encapsulated in the soft elastomer by pouring the silicone rubber in an octagonal mold and placing the dome in it. In this way the dome is fully encapsulated in the elastomer. The top and bottom electrodes are patterned on a flame-retardant substrate using printed circuit board (PCB) manufacturing technology. The top and bottom electrode plates, dielectric elastomer layer and PLA based dome are aligned and assembled using a cyanoacrylate glue. The total cost of all the components used in the sensor fabrication is US $10 which renders the proposed tactile force sensor low-cost and suitable for applications where frequent replacement of sensors is required.

### FEM analysis

**Fig 4:**
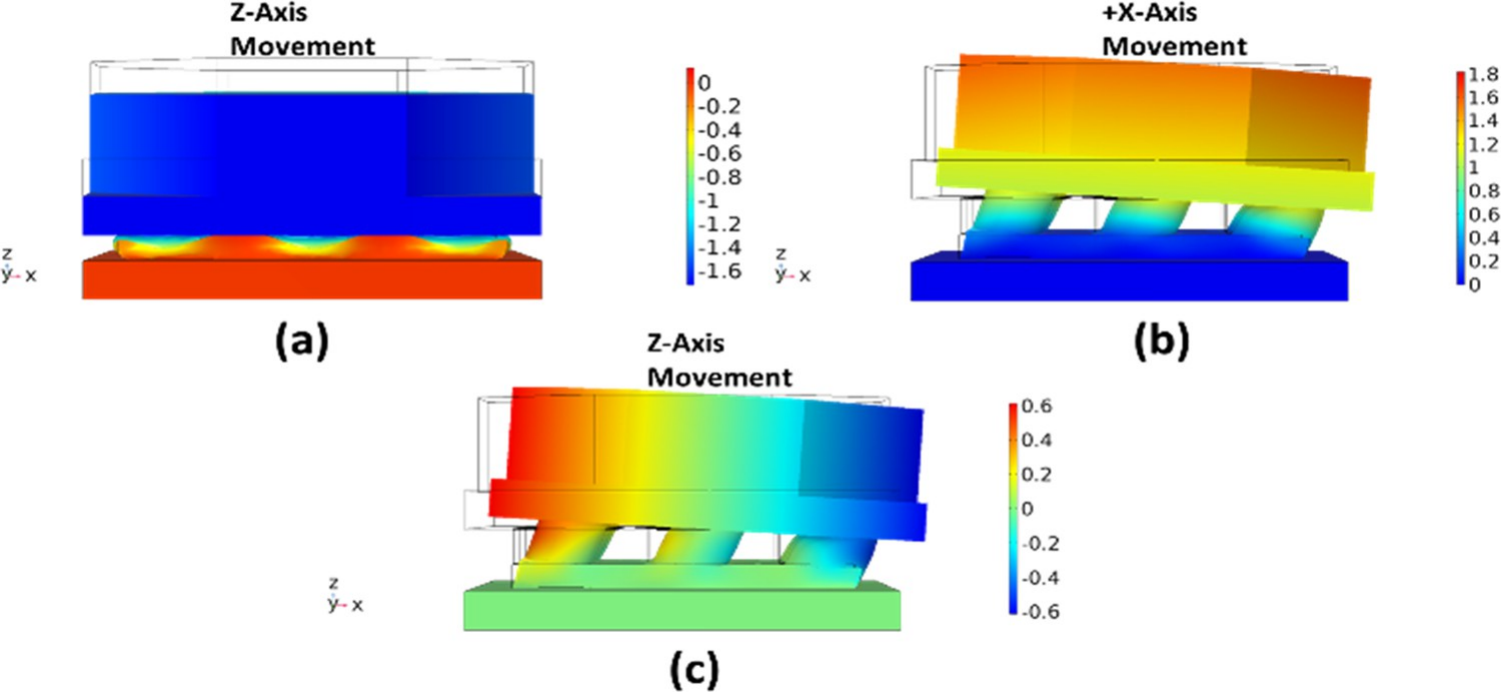
The displacement profile of the top electrode plates. *(a) Applied force in the* z-*axis*. *(b) applied force in the* +x-*axis*. *(c) undesired displacement of the top electrode plate (i*.*e*., *in* z-*axis) for an input shear force in the* +x-*axis*.

The working principle of the sensor, with respect to the applied force in normal and shear axis, is analyzed through finite element method (FEM) simulations in COMSOL Multiphysics. A density of 1100 kg/m3, Young’s Modulus of 0.05 MPa and Poisson’s ratio of 0.49 is assumed for RTV-528 silicone rubber in the FEM analysis (Vaicekauskaite et al. [24]). Fig 4(A) and 4(B) show the displacement profile of the top plate of the sensor for an applied normal and +x-axis shear force respectively. The results show that for an applied force in the z-axis, the top plate moves uniformly downwards. For an applied +x-axis shear force, the top plate moves in the axis of the applied force. However, there is an undesired out-of-plane deflection. Fig 4(C) shows that the undesired deflection in the z-axis for the top plate corresponding to a +x-axis shear force is symmetric with respect to the center. The z-axis deflection in the left and right side of top plate are equal with respect to the plate center.

**Fig 5:**
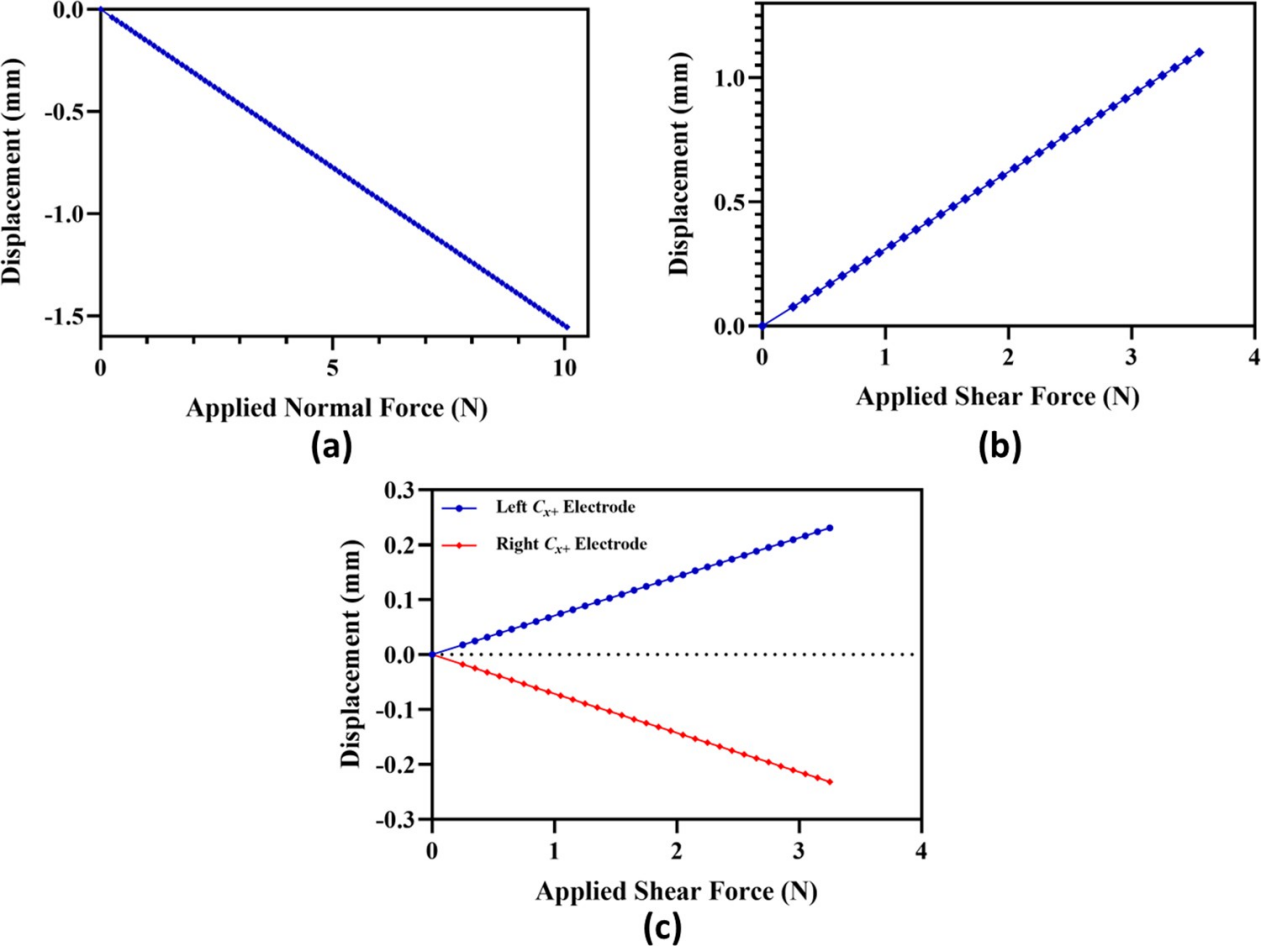
*FEM simulation results (a) Downward displacement of the top plate for applied normal force*. *(b) Shear displacement of the top electrodes plate for applied shear force (+*x*-axis)*. *(c) Undesired displacement of top electrodes (*C_X+_ *Capacitor) for applied shear force (+*x*-axis)*.

Fig 5(A) shows the displacement in the top plate of sensor with respect to the applied force in the z-axis. The results show that the displacement in the plate increases linearly in z-axis direction with an increase in the force. For an input force of 10 N the top plate transverses 1.55 mm in downward direction through the available gap of 2.5 mm between the top and bottom plate of the sensor. Fig 5(B) shows the displacement in the top plate in the +x-axis for an input +x-axis shear forces up to 3.2 N. The displacement profile is linear and at 3.2 N the displacement in the top plate is 1 mm. The clearance between the electrodes for the proposed sensor is 1 mm, thus after 1 mm displacement the capacitance change in the +x-axis electrodes will reach its maximum value. The undesired z-axis displacement in the capacitance electrode pairs for the applied +x-axis shear forces up to 3.2 N is shown in Fig 4(C). The results show that the undesired displacement is symmetric in the C_x+_ electrode pairs with a maximum value of 0.252 mm at 3.2 N as shown in Fig 5(C).

## Experimental characterization

### Experimental setup

**Fig 6:**
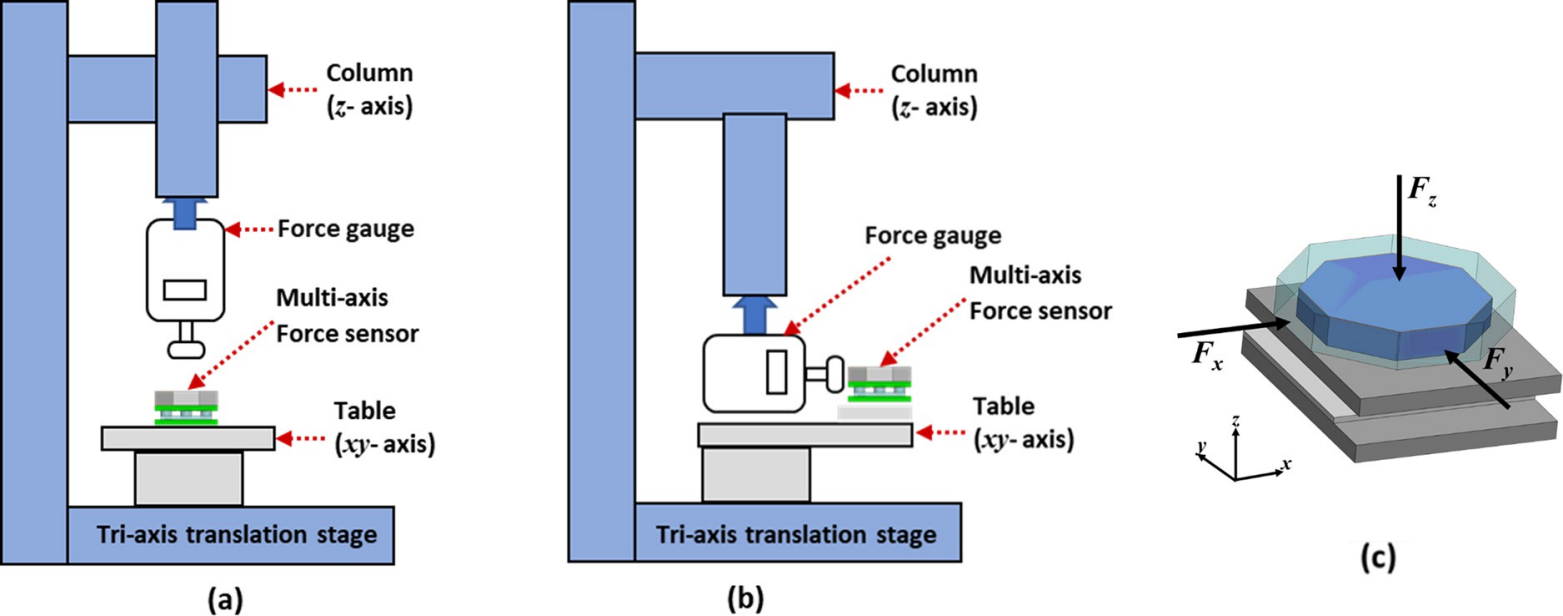
Schematic of tri-axis translation stage (a) Normal force configuration (b) Shear force configuration. (c) Illustration of the applied normal and shear axes forces.

Fig 6(A) and 6(B) shows the experimental setup implemented for the testing of the tactile force sensor. The sensor is tested by mounting it on a three-axis translational stage of a computerized numerical control (CNC) milling machine. The sensor is installed on the xy translation table of the machine, and a digital push-pull force gauge attached to z-axis moving stage is used to apply force on the sensor. The machine has a displacement resolution of 1 μm. The digital push-pull force gauge has a force range of 50 N with resolution of 100 mN is used to measure the applied force on the sensor. The machine is controlled manually through a knob on its control panel for static loading and the applied force is measured through the digital force gauge. The schematic for the force application on the top octagonal dome of the sensor for both shear and normal forces is shown in Fig 6(C). The sensor is shielded to reduce the effects of electromagnetic interference, providing a more stable output. A layer of thin copper film is used below the bottom plate as well as between top plate and dome while performing the experiments. The copper film is connected to the ground terminal of the sensor. The dome above the sensor serves the purpose of facilitating easy installation in various environments. The dome directly interacts with the surroundings, providing protection against unwanted interferences for the sensor.

**Fig 7:**
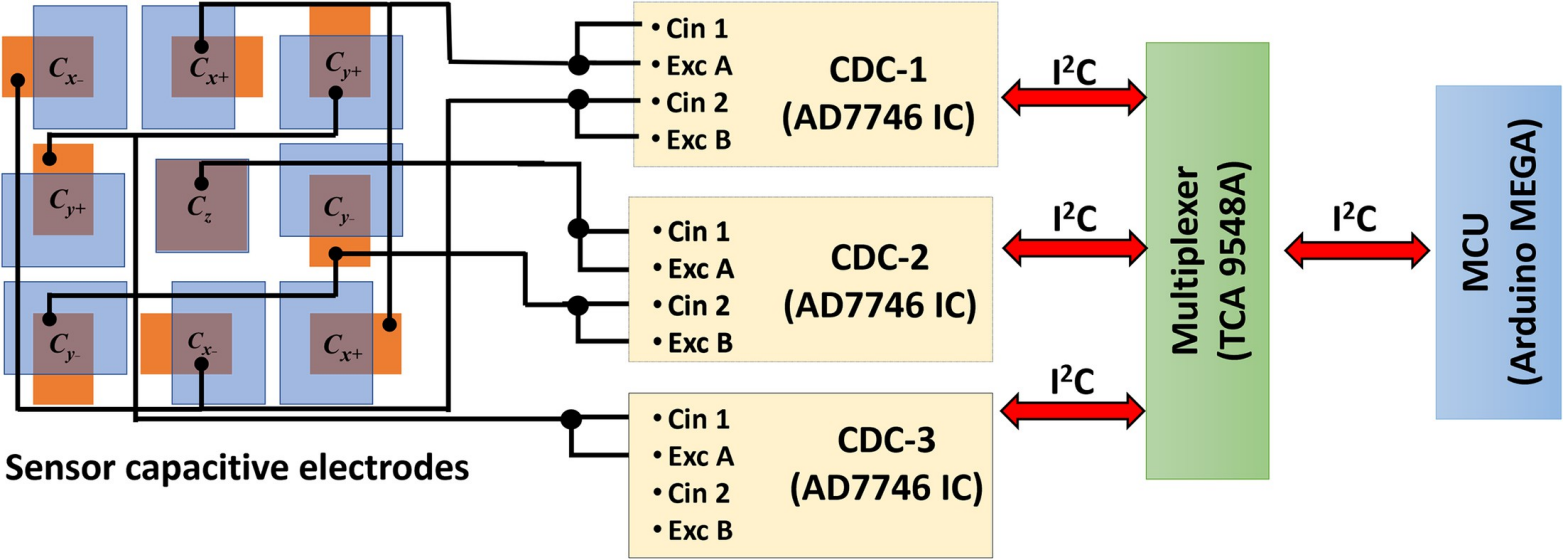
Data acquisition system implemented for the tactile force sensor characterization.

**Fig 8:**
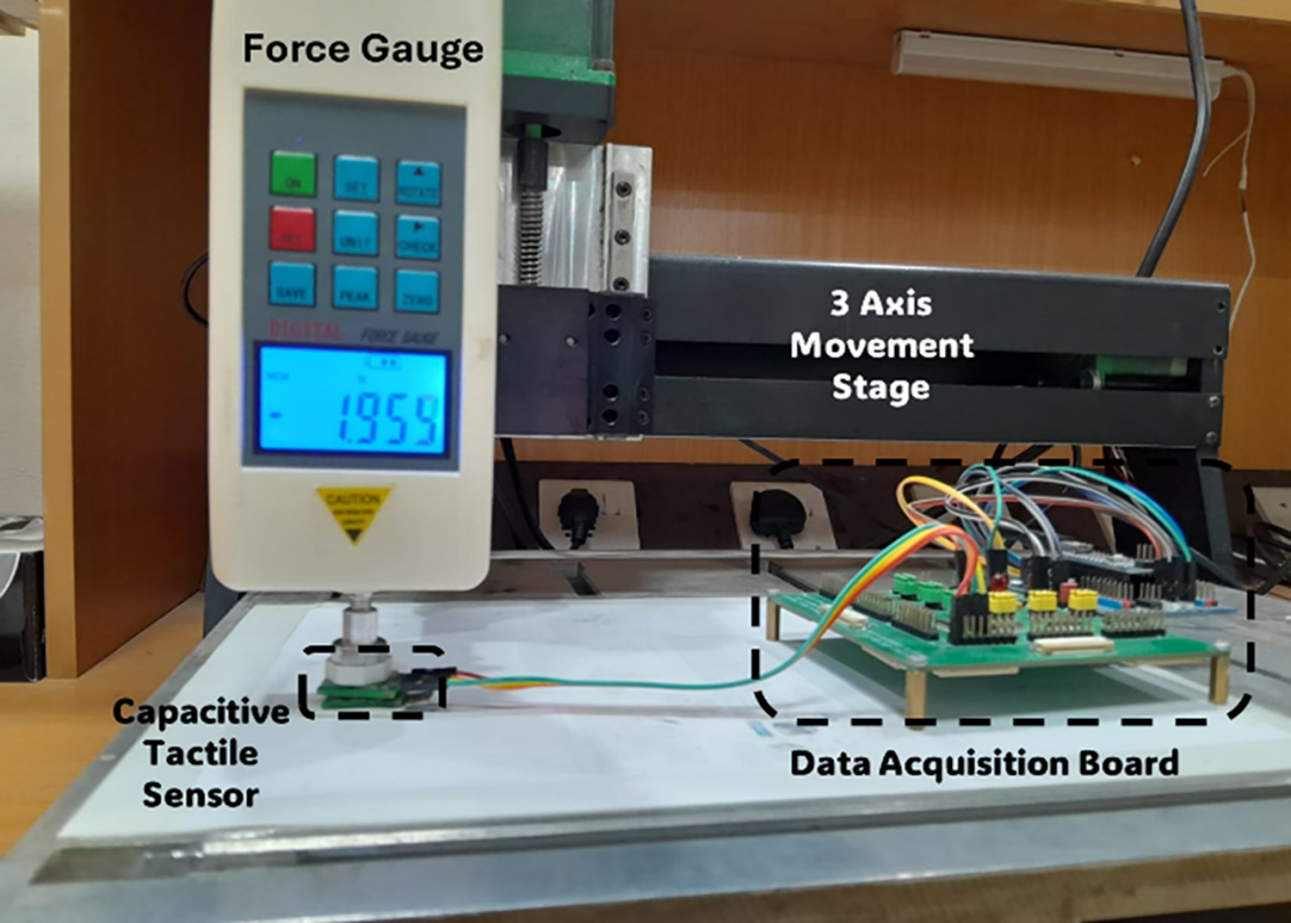
Photograph of experimental setup used for testing the sensor.

For data acquisition from the capacitive tactile sensor, a customized board based on AD7746 capacitance to digital converter (CDC) is used. The CDC is a sigma-delta modulation based dual channel IC which can measure both the floating and differential capacitance values. The CDC IC has a full-scale capacitance measurement range of ±4 pF with a resolution 4 aF. For the tactile force sensor data acquisition, a total of three AD7746 CDC ICs are used along with a multiplexer and microcontroller to acquire the data from the five capacitance electrode pairs. Fig 7 shows the schematic of the data acquisition system for the tactile force sensor. Fig 8 shows the experimental setup used to get the results.

### Experimental results

**Fig 9:**
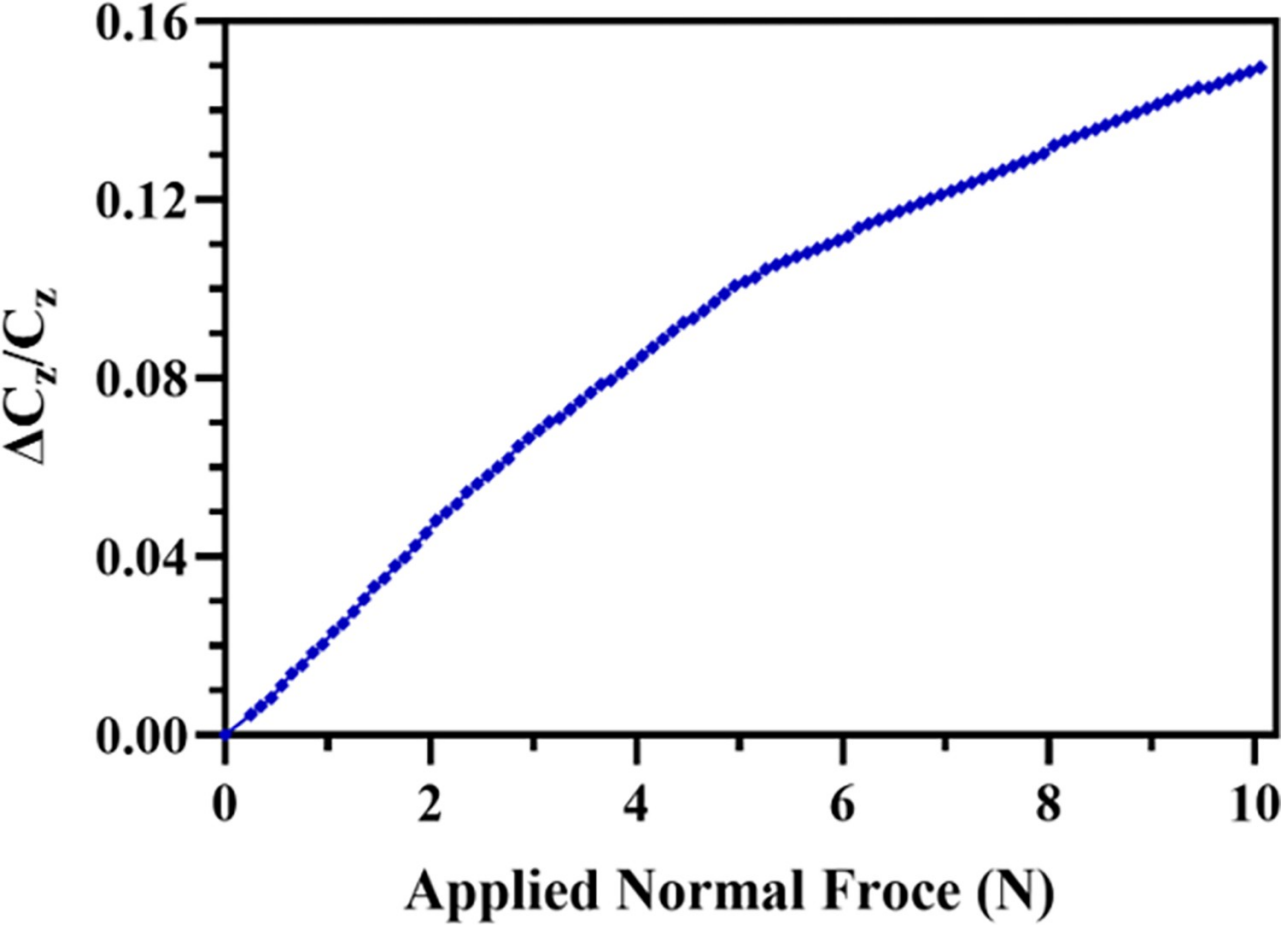
*Normalized capacitances change in the* z-*axis with an increase in the applied normal force*.

Fig 9 shows the normalized capacitance change *(*Δ*C*_*z*_*/C*_*z*_*)* in the capacitive electrode pair *C*_*z*_ of the sensor for an applied normal force (*F*_*z*_) in the *z-*axis with a step size of 0.1 N. The results show that with an increase in the applied force the normalized capacitance change increases due to a decrease in the distance between the electrodes. The output response curve shows two distinct regions; (a) in the force range of 0 to 5 N, the output capacitance of the sensor increases linearly with an increase in the force and (b) in the force range of 5 N to 10 N, again the capacitance change is linear but rate of change of normalized capacitance change is much less in comparison to the 0 to 5 N input force range.

This difference can be attributed to the nonlinear electrostatic force between the electrodes and hyperplastic behavior of the RTV-528 silicone rubber elastomer between the two electrodes. After the maximum input force of 10 N, the output of the sensor saturates since the elastomer has reached its maximum deflection limit. The ratio of relative change in capacitance to change in force provides the sensitivity of the sensor. The percentage sensitivity is given by S=(ΔC/(C.ΔF))×100. Here *ΔF* is the total change in applied force. The sensitivity of sensor for the force range of 0 to 5N and 5 to 10 N is 2.03%/N and 1.02%/N respectively.

**Fig 10:**
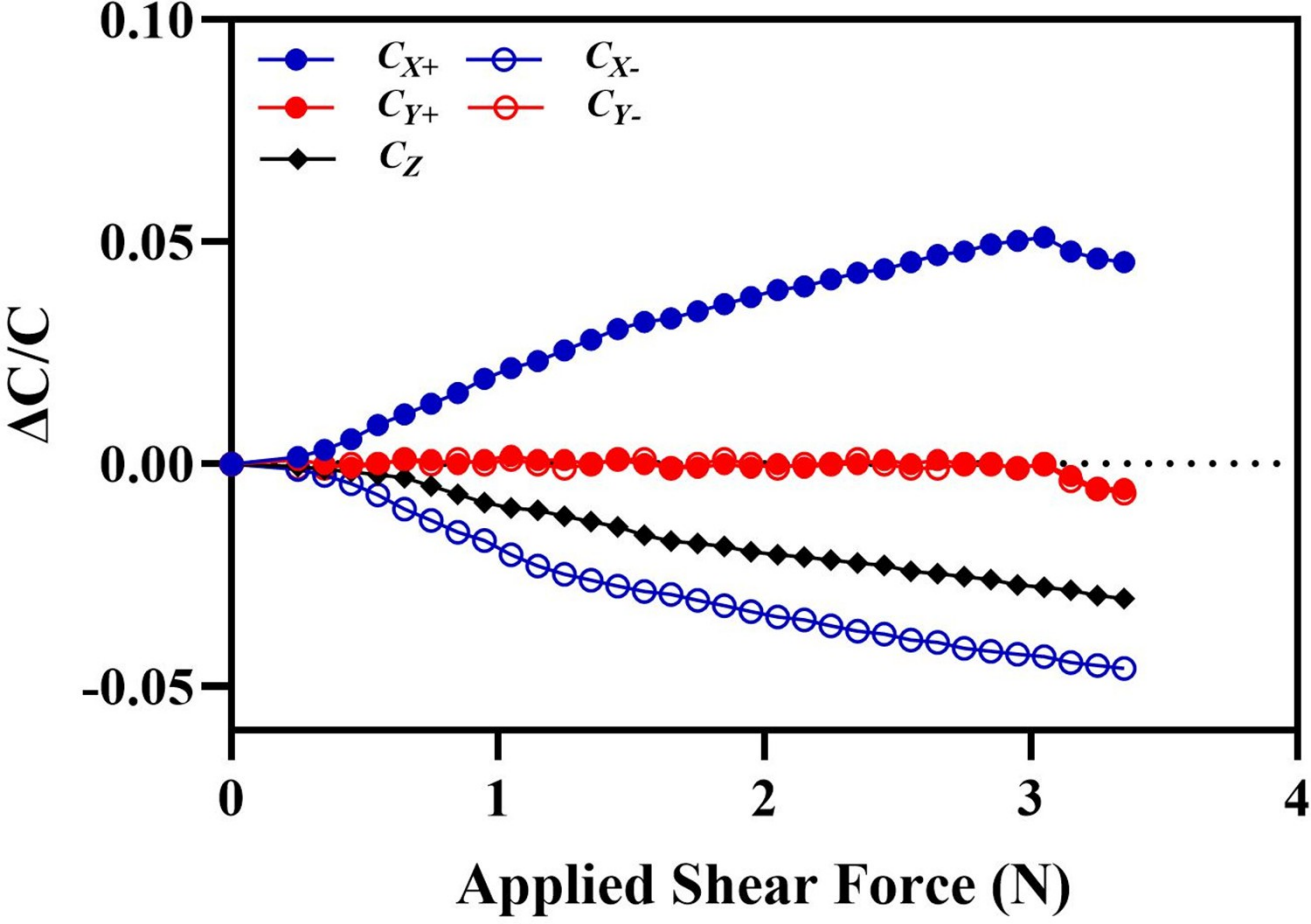
Normalized capacitances change in the sensor electrode pairs for an input force in the shear +x axis.

For the characterization of the output response of the sensor corresponding to an input shear force, a force (*F_x_*) is applied in the +*x*-axis of the sensor dome. Fig 10 shows the normalized capacitance response of all the five capacitors of the sensor for input shear force in the +x axis. The results show that for an input force in the +*x*-axis, the output capacitance of the capacitive electrode pair *C*_*x+*_ increases, for electrode pair *C*_*X₋*_ and *C*_*Z*_ decreases and remains constant for the electrode pairs *C*_*Y+*_ and *C*_*Y-*_. These results are consistent with the sensor shear force sensing scheme presented in Table 1. The normalized change in capacitance for the sensor electrode pairs *C*_*x+*_ and *C*_*x−*_is linear and with equal value for a input force up to 3.1 N. For an applied force above 3.1 N, the change in capacitance in the electrode pairs *C*_*X+*_ and *C*_*X-*_ starts decreasing. This is because at this force the top electrode of the electrode pairs *C*_*x+*_ and _*₋*_ *C*_*x−*_ has moved the maximum allowed displacement of 1 mm which is the initial clearance between the in-plane electrodes and displacement above 1 mm leads to decrease in capacitance due to decrease in the overlapping area. This result is also verified by the FEM simulation results presented in Fig 5(B) where the displacement value of 1 mm is estimated for an applied force of 3.2 N in the +x-axis. The sensitivity of the sensor calculated to be 1.65% /N for an input shear force in the range of 0 to 3.1 N with relative normalized maximum capacitance change of 5.1% at 3.1 N. Since the configuration of the electrode pairs in the tactile force sensor for the shear force measurement is symmetric, it is assumed that output of the sensor will follow the similar values for the ±y-axis shear forces.

### Force calibration

For the use of the tactile force sensor in the robotic surgical systems, it is essential that the sensor output capacitance change be calibrated with respect to both the normal and shear forces to calibrate the sensor and achieve a correlation between the change in capacitance and applied forces. A second order polynomial is fitted on the output capacitance change curves and corresponding coefficients are derived to obtain the following equations.

$$\Delta C_{s}=-0.0029{\;F}^{2}+0.0268\;F+0.0031$$ (3)

$$\Delta C_{N}=-0.0011\;F^{2}+0.0253\;F+0.0008$$ (4)

**Fig 11:**
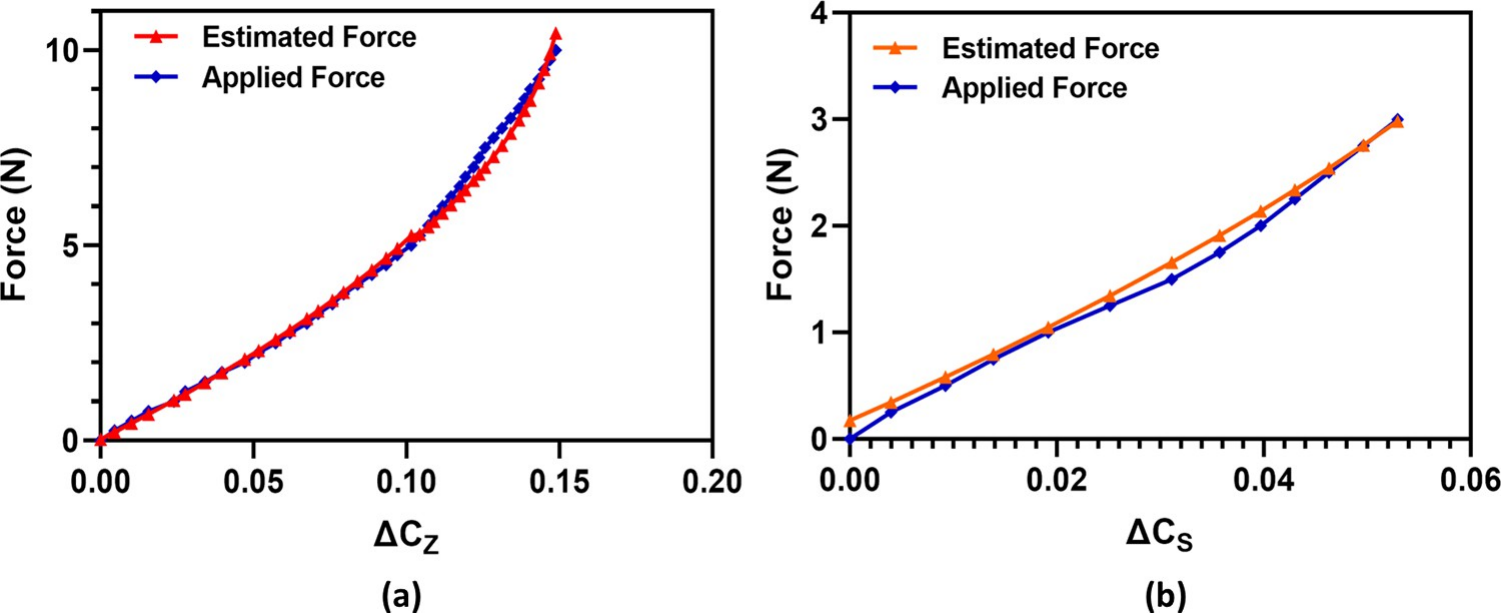
Comparison of the actual applied force and estimated force to the sensor (a) Normal (z-axis). and (b) Shear +x-axis.

Where Δ*C*_*S*_ and Δ*C*_*N*_ are the measured change in capacitance values for the applied shear and normal force to the sensor respectively while F is the estimated force from the measured capacitance change values. The validation of these mathematical expressions is performed by applying a range of known input forces to the tactile force sensor, with a step size of 0.25 N, in the normal and shear axis and recording the output capacitance change values. The capacitance change values are then used as an input to the Eq (3) and (4) to estimate the input force. Fig 11(A) and 11(B) show the comparison of the actual applied force to the sensor and corresponding estimated force for both normal and shear axis respectively. There is a close correspondence between estimated and applied force with maximum error of only 5% for the normal force and 5.8% for the shear force estimation.

## Discussions

**Fig 12:**
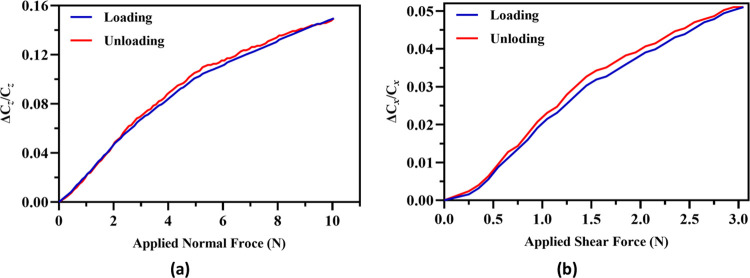
*The output normalized capacitance changes of the sensor for a loading-unloading cycle in a normal axis and b shear +*x *axis*.

The proposed tactile force sensor has RTV-528 silicone rubber elastomer as a dielectric layer between the two electrode plates. As stated by Feng et al. [25] one of the mechanical properties of the elastomers for tactile force sensor is their viscoelastic behavior which may lead to hysteresis. The hysteresis error for the tactile force sensor is estimated by applying a loading-unloading cycle on the sensor in both normal and shear axis as shown in Fig 12(A) and 12(B) respectively. The force in both loading and unloading cycle is changed by a step size of 0.1 N. The results show that the capacitance value for the unloading curve is slightly higher in comparison to the loading curve. This higher value of capacitance during unloading can be attributed to the stress relaxing in the RTV-528 silicone rubber elastomer. A hysteresis error of 4.94% in normal direction and 4.69% in shear axis is obtained based on the measured data.

**Fig 13:**
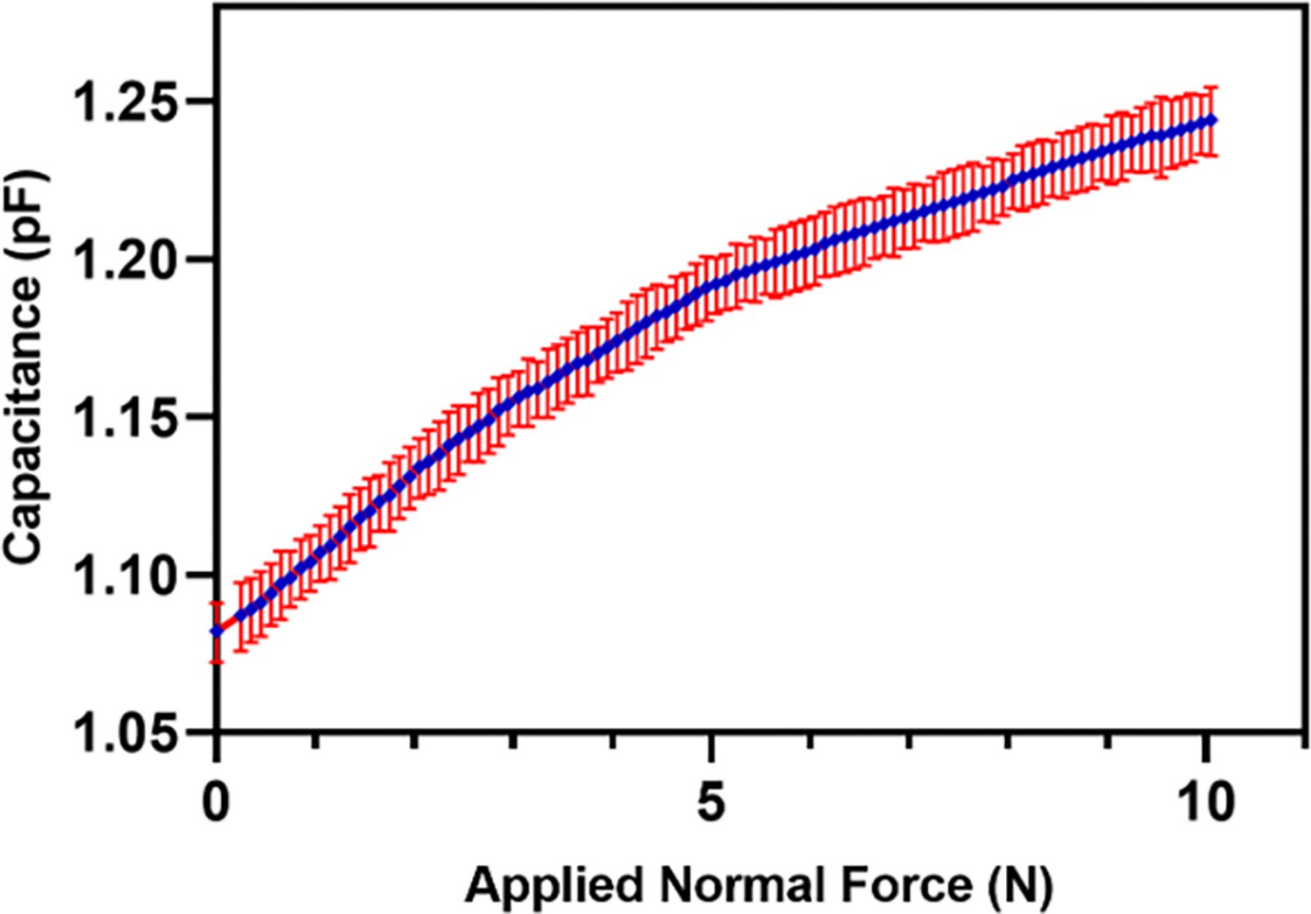
Output capacitance of the sensor against normal force for five loading cycles.

The repeatability of the proposed sensor is analyzed by applying five loading cycles in the normal axis with an interval of 10 minutes. The capacitance of *C*_*z*_ capacitor is recorded for each cycle. Fig 13 shows the plot of the average value of the measured capacitance for the five loading cycles with standard deviation error for each input force in the range of 0 to 10 N force. The sensor shows good signal stability by exhibiting a small repeatability error of 4.81%.

**Fig 14:**
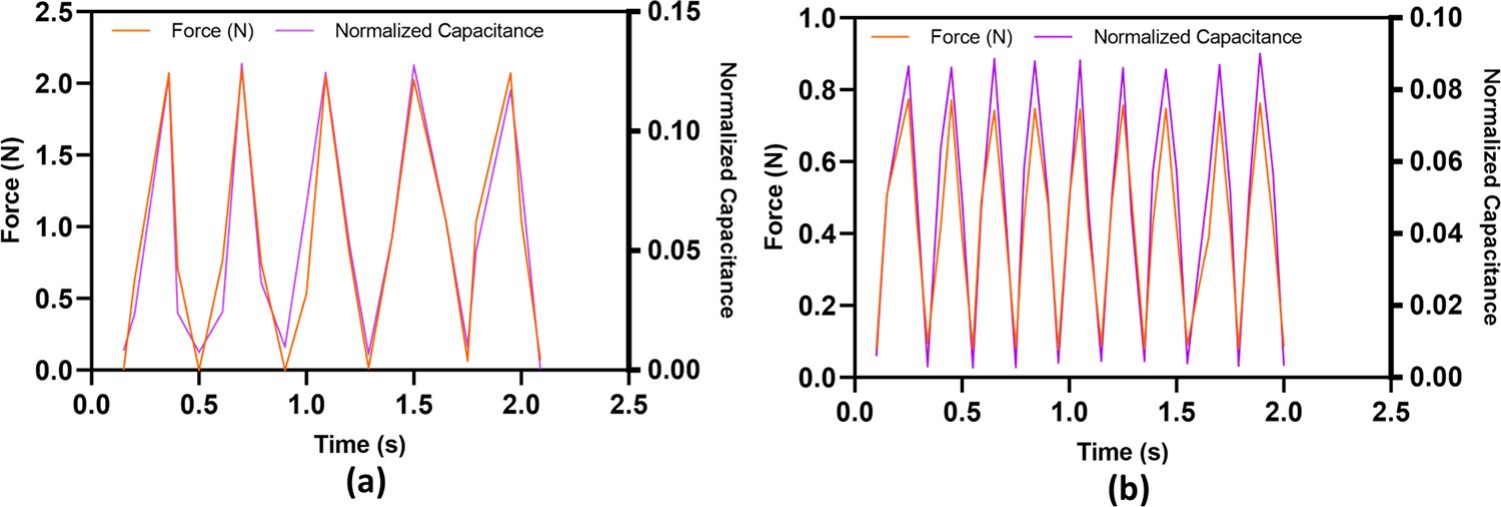
Dynamic response of the sensor for an input normal of (a) 2.5 Hz and (b) 4.5 Hz.

The dynamic response of the sensor is obtained by loading and unloading the sensor with a dynamic input force having a frequency of 2.5 Hz and 4.5 Hz. The result of the dynamic testing is shown in Fig 14. The results indicate that the change in output capacitance correlates with the applied input force. The slight variation in the amplitude of the input force across different cycles is likely due to slight play in the tri-axis translation test bench. The output response of the sensor for both input forces closely resemble the static input force. The minor difference is caused due to the viscoelastic behavior of dielectric elastomer pillars. The sensor shows precise response at both input frequencies, suggesting that it is quite suitable for laparoscopic MIRS applications, where the typical grasping frequency is below 3 Hz [26, 27]. The experimental setup employed for dynamic testing of the sensor has a constraint to operate on lower frequencies. This limitation has restricted our ability to test the sensor at frequencies exceeding 5 Hz.

**Fig 15:**
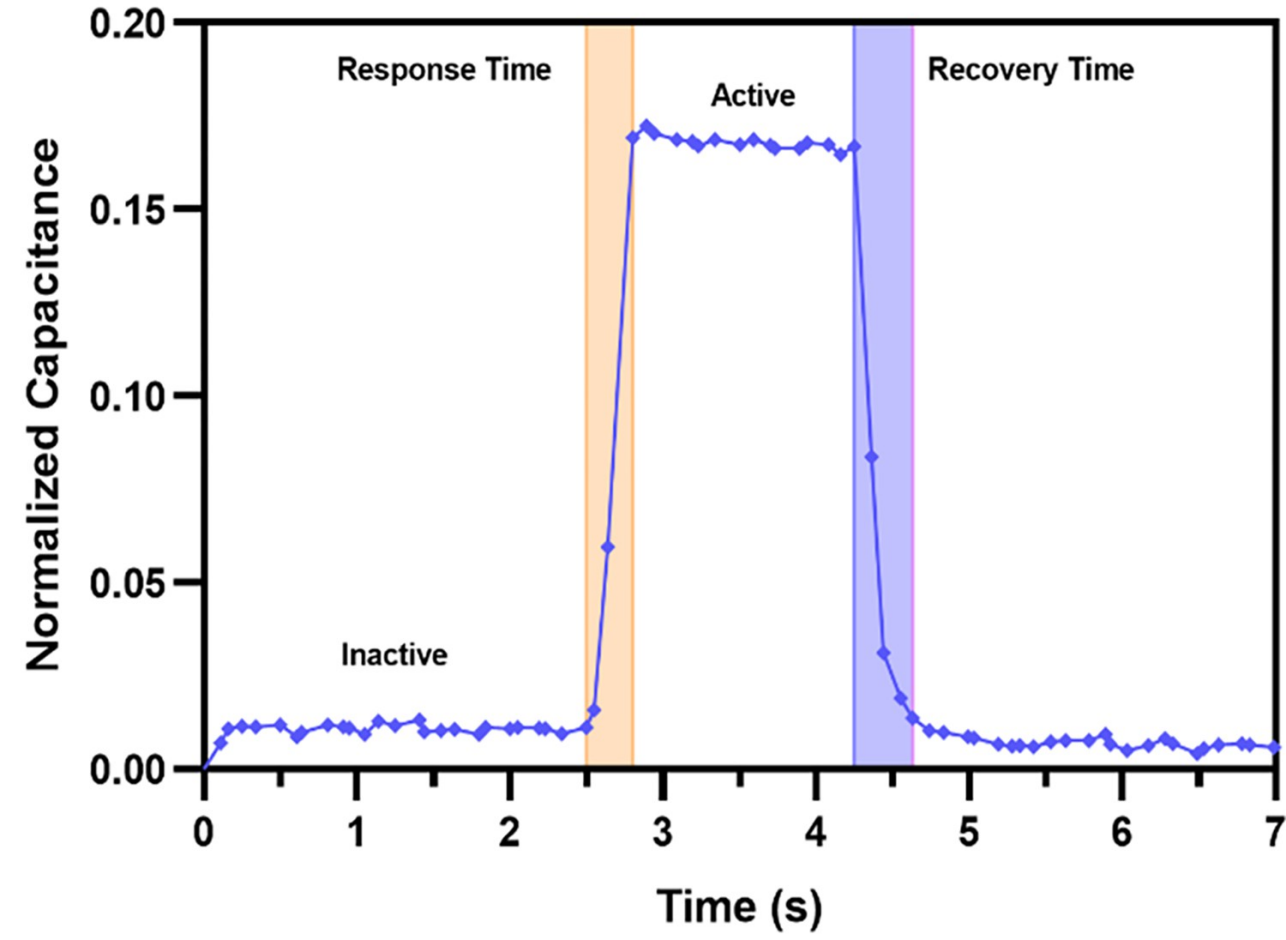
The force response curve for normal applied force of 1.5 N at 2 Hz.

A higher signal to noise ratio (SNR) is desired for better accuracy and high sensitivity of a sensor. The step response of the sensor is recorded to analyze the signal to noise ratio (SNR) of the sensor. Fig 15 shows the force response curve when a normal force of 1.5 N is applied on the sensor for two seconds and then removed. The average noise capacitance for inactive regions is found to be 1.954 f F. The value of SNR is calculated by, $SNR=20\;{log}_{10}\left\{\frac{C_{A}-C_{I}}{N_{I}}\right\}$. Here, *C*_*I*_ is average inactive zone capacitance, *C*_*A*_ is average active zone capacitance and *N*_*I*_ is total inactive noise signal. The sensor exhibits an SNR value of 28.13 dB at 2 Hz operating frequency. The sensor has a response time of 0.3 seconds and recovery time of 0.38 seconds at 2 Hz operating frequency. The low frequency constraint of the force application setup has limited the input frequency thereby increasing the response and recovery time.

**Fig 16:**
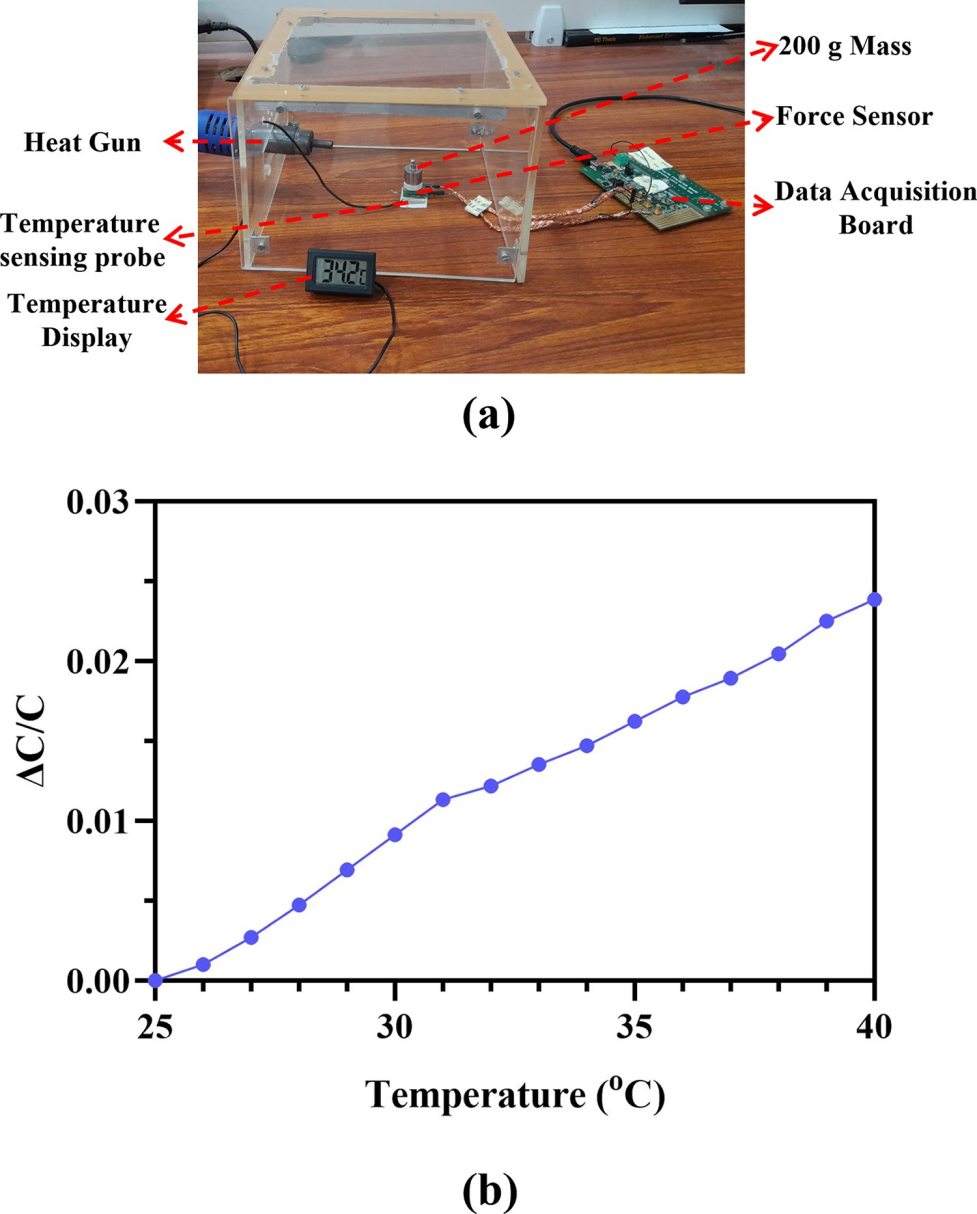
The effect of varying atmospheric temperature (a) Experimental setup. (b) Temperature vs Normalized capacitance across a temperature range of 25–40°C.

To evaluate the sensor’s performance under varying atmospheric temperature, it was placed inside an acrylic temperature chamber. A heat gun was used to raise the temperature, and the chamber’s temperature was monitored by positioning the probe of a digital thermometer near the sensor. The experimental setup is illustrated in Fig 16(A). To measure the capacitance variation, a fixed mass of 200 g was applied on the sensor dome, exerting a downward force of 1.96 N along the Z-axis. The sensor’s capacitance was recorded across a temperature range of 25°C to 40°C, with readings taken at 1°C intervals. Fig 16(B) shows the plot of temperature versus normalized capacitance. The results indicate a slight increase in capacitance with rising temperature, with a 2.4% change in nominal capacitance at 40°C compared to 25°C. The temperature sensitivity of the sensor is 0.94 fF/°C.

**Fig 17:**
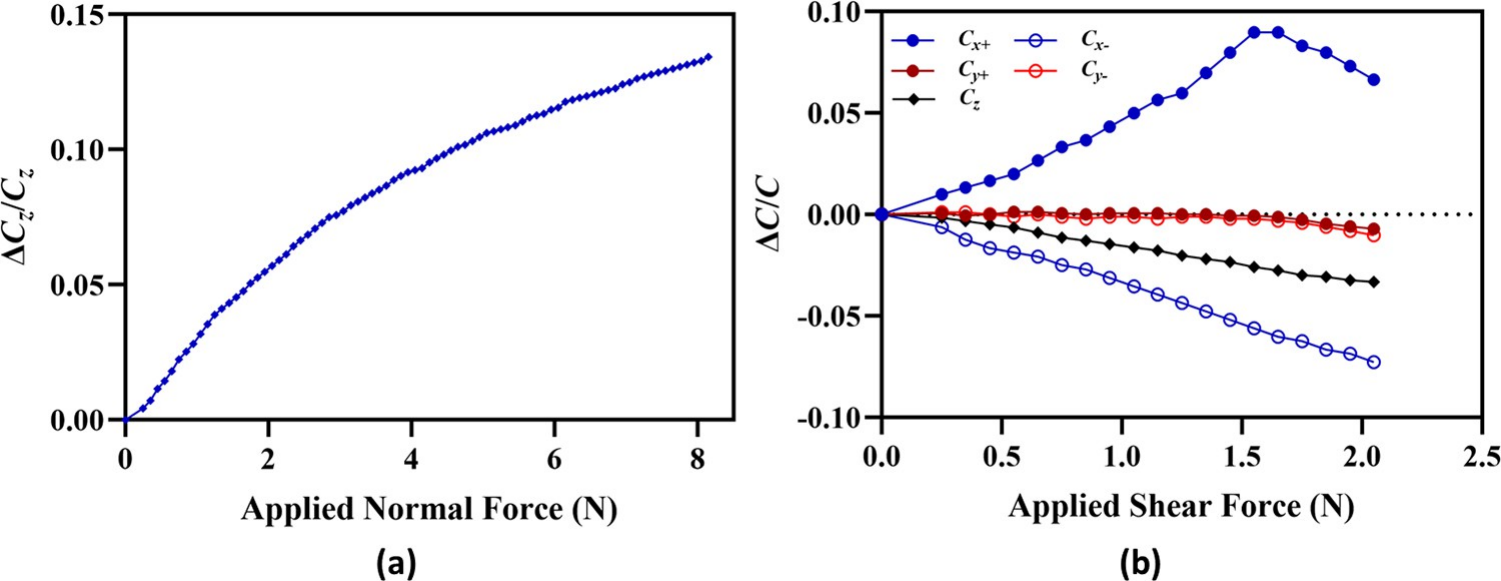
Normalized capacitances change in the sensor, with Ecoflex 00–30 dielectric elastomer, for an applied (a) Normal force (b) Shear force in the +x-axis.

Since the proposed tactile force sensor is low cost due to rapid prototyping, we have fabricated another sensor by replacing the RTV-528 silicone rubber with silicone Ecoflex 00~30 as a dielectric elastomer between the two parallel electrode plates. The sensor is tested for both the normal and shear axis input force. The results in Fig 17 show that for the sensor with Ecoflex 00~30 as an elastomer, the measurement force range for both normal and shear axis decreases to 8.2 N and 1.5 N respectively. However, the sensitivity value for the normal axis is 1.65% /N and for shear axis is 5.79% /N which are higher than that obtained for the sensor with RTV-528 silicone rubber as dielectric elastomer. The lower force range and higher sensitivity for the Ecoflex 00~30 dielectric elastomer sensor can be explained with lower value of mechanical stiffness of the Ecoflex 00–30 dielectric elastomer.

In tele-manipulation based robotic surgical systems, the estimation of surgical tool-tissue interaction forces is required to avoid damage to the tissues. Golahmadi et al. [28] reported that a large range of surgical tool interaction forces exists depending on a particular surgical procedure varying from 0.04 N for ophthalmic surgery to 210 N for orthopedic surgery. The input force measuring range of 0 ~ 10 N for the proposed tactile sensor makes it suitable for measuring the tool-tissue interaction and grasping forces in laparoscopic MIRS where the forces are in the range of 4.5 N.

Table 2 compares various capacitive tactile force/pressure sensors, focusing on two key parameters: sensitivity and measurement range for both the normal and shear axes. The analysis reveals a distinct trade-off between these two parameters. Sensors with higher sensitivity typically exhibit lower measurement ranges, making them ideal for applications where detecting small changes in force is crucial, but the overall forces involved are relatively low. Conversely, sensors with larger measurement ranges generally have lower sensitivity, making them more suitable for environments where larger forces need to be measured, and small force variations are less critical. This comparison highlights the importance of selecting the appropriate sensor design based on the specific requirements of each application. Ultimately, the balance between sensitivity and measurement range depends on the application. The proposed sensor, with its high sensitivity and moderate measurement range, offers an optimal compromise for applications that require both precision and the ability to handle mid-range forces, making it highly adaptable for various robotic and tactile sensing tasks.

**Table 2:** Comparison of sensitivity and measurement range in normal and shear axis from different studies.

Ref	Type	Axis	Normal Force/Pressure		Shear Force/Pressure	
			Sensitivity	Measurement Range	Sensitivity	Measurement Range
[12]	Capacitive	3 Axis	1.3%/mN	20 mN	1.2% /mN	20 mN
[13]	Capacitive	3 Axis	0.024%/kPa	10 kPa	0.00028%/kPa	220 kPa
[14]	Capacitive	3 Axis	0.583%/N	0.5 N	0.583%/N	0.5 N
[15]	Capacitive	3 Axis	1.08%/N	0.7 N	1.2%/N	0.6 N
[16]	Capacitive	3 Axis	0.04%/N	15 N	0.03667%/N	15 N
[17]	Capacitive	3 Axis	0.0028%/N	50 N	0.0061%/N	10 N
[18]	Capacitive	3 Axis	0.0378/N	5 N	0.018/N	1.5 N
This study	Capacitive	3 Axis	2.03%/N	10 N	1.67%/N	3.1 N

Palpation is a simple but important physical examination to estimate the size and nature of an organ of human body. Reliable tactile feedback in minimally invasive robotic surgeries can be crucial for achieving better clinical outcomes. Robotic probes with integrated sensors can efficiently perform palpation examinations and provide better outcomes. One such study is reported by Konstantinova et al [29]. They used a robotic probe to examine silicon tissue phantom with artificial nodules. They used a model extracted from human perception and reported enhanced perception results as compared to static indentation.

**Fig 18:**
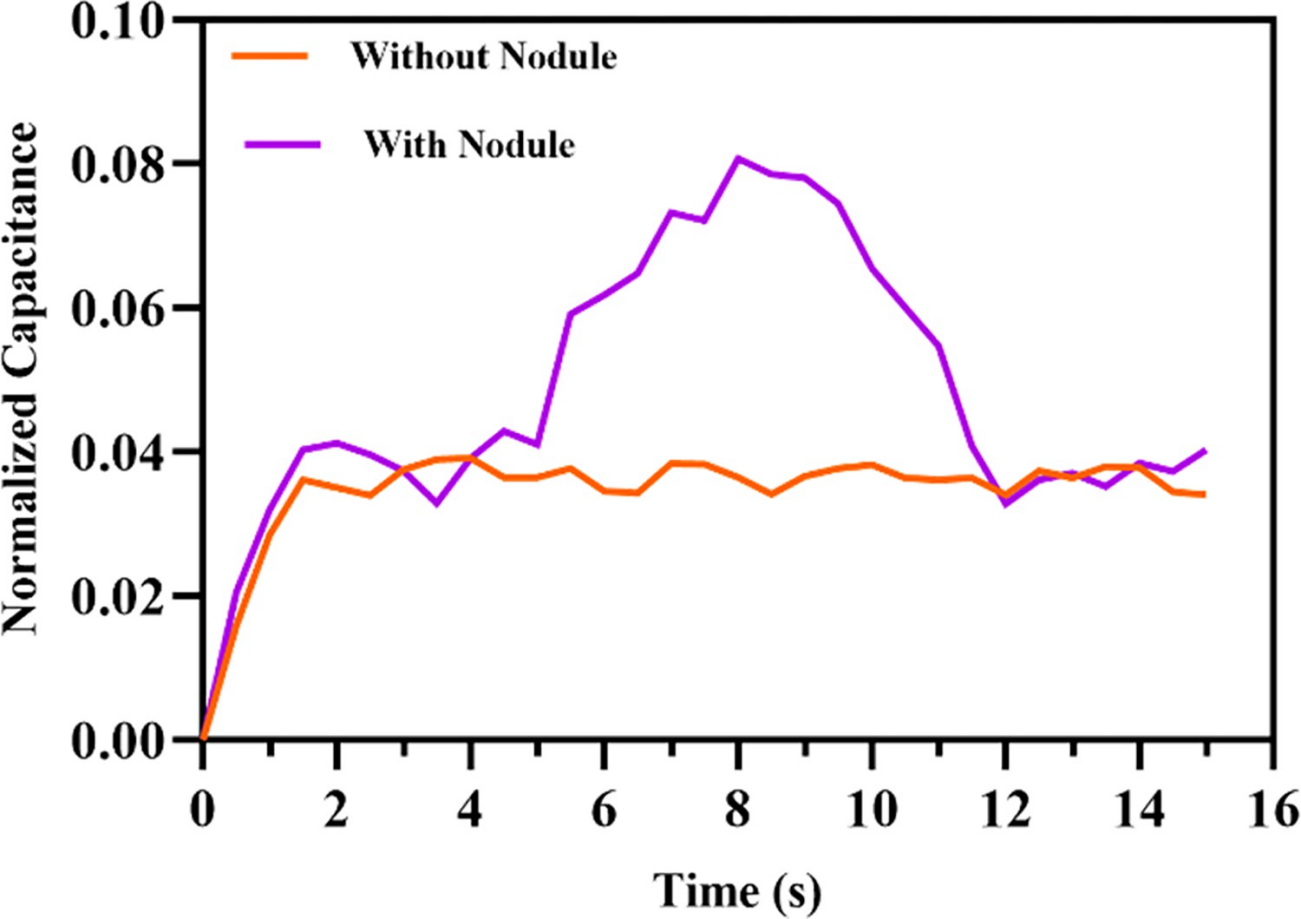
Sensor output for nodule detection.

**Fig 19:**
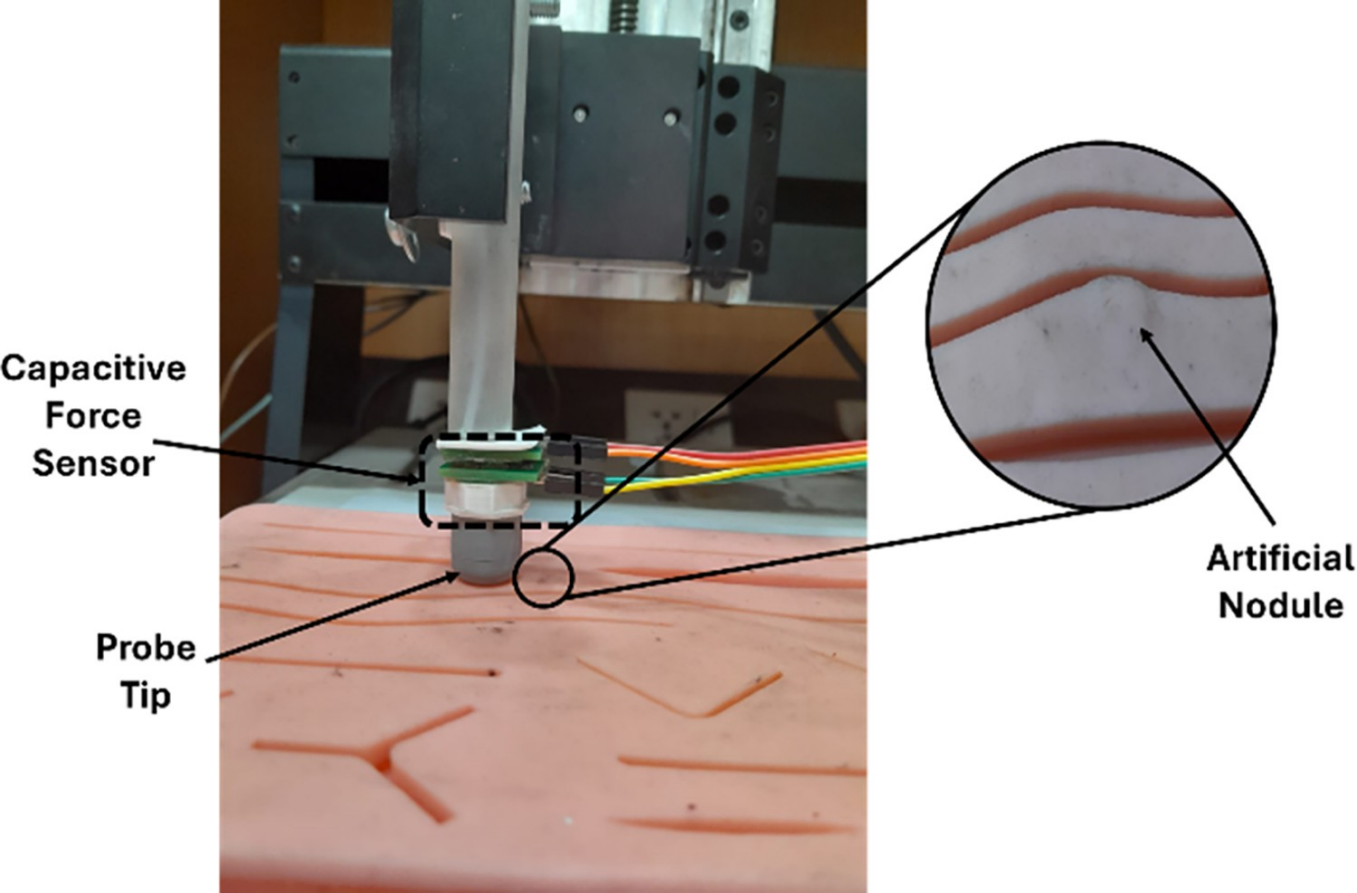
Picture of experimental setup for nodule detection.

To demonstrate the utility of reported capacitive force sensor for tactile feedback a simple test is performed. A silicone pad from suture practice kit is utilized for this purpose. An artificial nodule of 5mm is used to perform the sample examination. The sensor is installed in a probe in inverted configuration. The probe is attached to the 3 Axis stage which is programmed to slowly sweep the silicone pad in horizantal direction initially without nodule. Then the same sweep is repeated with nodule. The output signal is recorded for both runs. The output is taken only from normal force electrodes for this experiment. Fig 18 presents the results of change in capacitance when there is no nodule and when a nodule is detected. The experimental setup for this experiment is shown in Fig 19.

The results show that, for the plain sweep without a nodule, the output signal varies slightly within a narrow range. Whereas, for the sweep with a nodule, the sensor generates a noticeable peak for the portion of nodule. This peak occurs due to the perturbation caused by the presence of nodule inside the pad. This indicates that the proposed sensor is capable to detect nodules through physical examination.

## Conclusion

A mesoscale three-axis capacitive tactile force sensor with high sensitivity, low cost and fully decoupled output response for input forces is designed and characterized in this work. The proposed sensor has the capability to measure normal and shear forces in *x*, *y* and *z*-axis and is fabricated using rapid prototyping technique. The sensor has a force measurement range of 0 ~ 10 N in *z*-axis and 0 ~ 3.1 N in shear axis. The input force estimated by the sensor is compared with the actual applied force and the difference obtained is less than 6% which proves the accuracy of the sensor. The sensor is tested for both hysteresis and repeatability with error values of less than 5%. Another, sensor protype is fabricated and tested by replacing the dielectric elastomer of RTV-528 silicon with Ecoflex 00~30 which showed that higher sensitivity can be achieved with Ecoflex 00~30 dielectric elastomer with reduced force range. The force response curve and dynamic response of the sensor indicate that the sensor is suitable for the dynamic input forces ranging up to 5 Hz. The low-cost fabrication process and achieved characteristics of high sensitivity, fully decoupled output response and reliability for the proposed tactile force sensor make it suitable to be used in robotic surgical systems for tactile force feedback.
